# The Etiology of IgE-Mediated Food Allergy: Potential Therapeutics and Challenges

**DOI:** 10.3390/ijms26041563

**Published:** 2025-02-13

**Authors:** Michelle Carnazza, Robert Werner, Raj K. Tiwari, Jan Geliebter, Xiu-Min Li, Nan Yang

**Affiliations:** 1 General Nutraceutical Technology, LLC, Elmsford, NY 10523, USAnan.yang@gnt-us.com (N.Y.); 2 Department of Pathology, Microbiology & Immunology, New York Medical College, Valhalla, NY 10595, USA; 3 Department of Otolaryngology, New York Medical College, Valhalla, NY 10595, USA; 4 Department of Dermatology, New York Medical College, Valhalla, NY 10595, USA

**Keywords:** food allergy, IgE, biologics, natural products, probiotics

## Abstract

Immunoglobulin E (IgE)-mediated food allergy has been dramatically increasing in incidence over the last few decades. The combinations of both genetic and environmental factors that affect the microbiome and immune system have demonstrated significant roles in its pathogenesis. The morbidity, and at times mortality, that occurs as the result of this specific, reproducible, but impaired immune response is due to the nature of the shift from a regulatory T (Treg) cellular response to a T helper 2 (Th2) cellular response. This imbalance caused by food allergens results in an interleukin (IL)-4 and IL-13 dominant environment that drives B cell activation and differentiation into IgE-producing plasma cells. The resulting symptoms can range from mild to more severe anaphylaxis, and even death. Current therapeutic strategies involve avoidance and broad symptom management upon accidental exposure; however, no definitive cure exists. This narrative review highlights how the elucidation of the pathogenesis of IgE-mediated food allergy resulted in the development of therapeutics that are more specific to these individual receptors and molecules which have been relatively successful in mitigating this potentially life-threatening allergic response. However, potential adverse effects and re-sensitization following the conclusion of treatment has urged the need for improved therapeutic methods. Therefore, given the understanding of their mechanism of action and the overlap with the mechanism of IgE-mediated food allergies, probiotics and small molecule natural compounds may provide novel therapeutic and preventative strategies. This is compelling, as they have demonstrated success in clinical trials and may provide hope to improve quality of life in allergy patients.

## 1. Introduction

Food allergy is a growing health concern as its incidence has increased dramatically in westernized countries over the last few decades. Affecting approximately 20 million Americans, food allergy is the result of a specific and reproducible immune response occurring upon exposure to a given food. In the United States, there are an estimated 8% of children and 6% of adults affected by food allergy [1].

**Figure 1:**
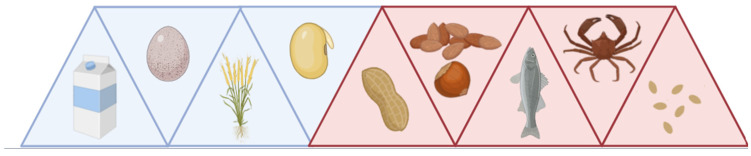
Common food allergies in the United States. Milk, egg, wheat, and soy are largely outgrown by adulthood (blue). Peanuts, tree nuts, fish, shellfish, and sesame are generally persistent and account for most fatal and near-fatal anaphylactic reactions (maroon).

Evidence suggests that the response of the body to specific allergens evolves over time, with the emergence and resolution of food allergies at all stages of life. While there have been 170 foods reported to cause an allergic reaction in persons in the United States, only 8–9 are responsible for 90% of them (Figure 1) [1,2]. Milk, egg, wheat, and soy constitute 85% of childhood food allergies that develop in infancy and are largely outgrown by 5 years of age [3,4,5]. However, persistent food allergies are seen with sensitivity to peanuts, tree nuts, fish, shellfish, and sesame, whereby only 2–20% will outgrow these allergies by adulthood [4,6,7]. Alarmingly, the persistent peanut and tree nut allergies account for most of the fatal and near-fatal anaphylactic reactions in the United States. Coinciding with this, studies have demonstrated not only an increase in food allergy prevalence but also increased persistence and severity.

Therefore, this narrative review aims to describe the current understanding of IgE-mediated food allergies, the advancements in knowledge of its etiology, and the consequential development of targeted therapies through the existing literature. With that, the limitations of targeted therapies of westernized medicine exist and, hence, natural products and probiotics that may serve as alternative therapies have been investigated. Given the understanding of their mechanism of action and the overlap it has with the mechanism of IgE-mediated food allergies, they may provide novel therapeutic and preventative strategies for patients in westernized countries.

## 2. Epidemiology

**Figure 2:**
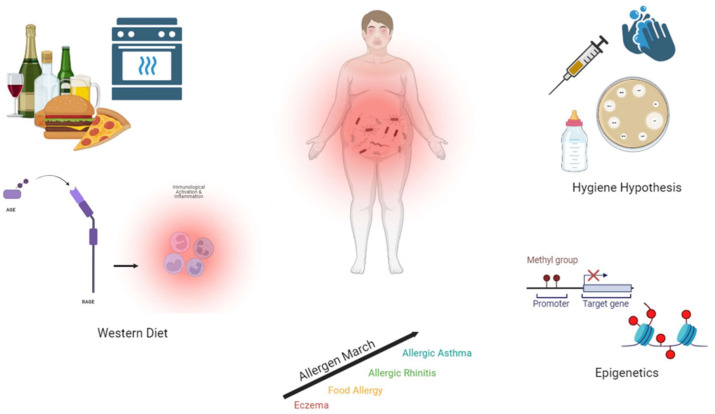
Factors contributing to increasing food allergy prevalence. Food allergy is hypothesized to be increasing in prevalence due to Western diet, hygiene, epigenetics, and the allergen march. These factors have a large influence on inflammation and the gut microbiome, which largely modulates the immune system.

Theories have been proposed for the increase in food allergy, termed the “second wave of the allergy epidemic”, including Western diet, the hygiene hypothesis, epigenetics, and allergen marching (Figure 2).

### 2.1. Western Diet

Regarding the Western diet, this is an unhealthy diet of processed foods and the rising obesity epidemic, resulting in lower life expectancy and lower quality of life [8,9]. This diet is one that is high in calories, saturated fat, salt, and sugars, with a very low diversity of healthy fruits, vegetables, and oils. With that, the Western diet is highly inflammatory and constitutes a risk factor for a variety of disorders, including food allergy [10]. This could be due to the decreased intake of micro- and macronutrients and the subsequent decrease in health-promoting gut microbiota metabolites. These metabolites are anti-inflammatory and protective from infections, contributing to gut integrity and immune homeostasis [6]. In stark contrast, the Mediterranean diet has been found to be protective against allergic disease, characterized by consumption of a large, diverse proportion of fruits and vegetables, olive oil, and fish [9]. These diets are rich in fats, indoles, and vitamins, which have benefits for intestinal homeostasis and immunity [11].

Western diets also include cooking food at higher temperatures, which is thought to play a role in the allergic response [12]. For example, in China, peanut consumption is like that of the United States, but the allergy rate is much lower. In China, peanuts are boiled, while in the United States, peanuts are roasted; therefore, it is thought that boiling may reduce the amount of allergen exposure. This highlights the false alarm hypothesis, whereby some foods, typically high in glucose, fat, and protein, when cooked in different ways, create specific products that mimic alarmins [12,13]. Advanced glycation-end products (AGEs), also called glycotoxins, are present when animal proteins and fats are cooked at high temperatures and expose amino acid chains that bind sugars. These alarmins can bind to their receptors, RAGE (receptor for advanced glycation end-products), which are highly expressed on dendritic cells, macrophages, T cells, and B cells. This RAGE activation results in downstream inflammation and promotes an adaptive immune response. Food allergy is therefore associated with these AGEs and proglycating dietary sugars which are priming innate signaling and causing allergy and inflammation [12,14]. In all, this demonstrates that the Western diet is a large contributor to the development of allergies.

### 2.2. The Hygiene Hypothesis

Early childhood exposure to microorganisms such as helminths and normal flora is thought to protect against allergies through immune system strengthening and education. The hygiene hypothesis contends that due to diminished immune-stimulating helminth and parasitic infections, the more sterile upbringing results in a less educated and experienced immune system [15,16,17]. Exposure to various microorganisms allows the innate immune system to learn the difference between harmful and harmless antigens. When immune system development is prevented in absence of pathogenic infections, it will begin to respond to harmless antigens instead.

In industrialized and developed countries, people typically live in the suburbs and cities rather than on farms, and grow up in cleaner, drier, dust-free homes composed of a smaller family size [16]. Children here are typically well vaccinated and have avoided allergenic foods in infancy, further promoting this diminished immune system activation [18]. Instead, guidelines support early, rather than delayed, peanut introduction [19]. Furthermore, in the United States, antibiotics are used more freely, which eliminates normal flora at the expense of eliminating pathogenic bacteria [16]. Moreover, babies are not breastfed as often as in developing countries and, instead, are given formula and processed foods devoid of healthy microbiota that could be passed through breastfeeding milk [17]. The fetal immune system undergoes significant development during pregnancy but is tightly regulated to tolerate maternal antigens and prevent excessive inflammation. Therefore, without this maternal influence of microbiota and passive immunity, the immune system remains underdeveloped. Other factors such as vaginal delivery, attending day care, and having pets are thought to be more protective against allergies through the sharing of microbiota [17,20].

Western culture also limits exposure to UV rays from the sun and is characterized by low vitamin D, which may also play roles in allergy development as the immunoregulatory and tolerogenic functions of vitamin D are lost [21,22]. This has been further evidenced as far as birth season and neonatal immune cell phenotypes. Winter and fall newborns present with high levels of activated T cells, IL-13, and IL-5 compared to summer newborns [23]. Therefore, Western culture and modern living prevent the immune system from learning to differentiate between the harmful and harmless antigens.

### 2.3. Epigenetics

Food allergy prevalence is not only owed to environmental risk factors described above, but also genetic risk factors. If one or two parents have allergies, this increases likelihood to 50% and 75% of their children developing allergies, respectively [2]. However, it has been demonstrated that classical genetics alone cannot explain the increased food allergy prevalence. It has been proposed that maternal allergy increases food allergy susceptibility through the gene–environment interaction, or epigenetic regulation, of cytokine expression by deoxyribonucleic acid (DNA) methylation, histone modification, and microRNA expression [24]. The DNA methylation profile of those with cow milk allergies, for example, is distinct from those without [25]. This has been extensively studied in the expression of IL-4, a key cytokine in allergic disease, and the epigenetic regulation of its promoter in the context of maternal peanut allergy [26]. Further, DNA methylation at cell cycle and MAPK signaling-associated genes during early CD4+ T-cell development and other immunological signaling pathways have been demonstrated [27,28,29]. There have also been significant associations with gene polymorphisms of human leukocyte antigen (HLA)-DRB1, DQB1, and DPB1 [26,30]. Other genes, including CD14, FOXP3, STAT6, SPINK5, IL-10 and IL-13, have demonstrated polymorphisms that affect allergy prevalence as well [26,30]. In summary, there have been associations between risk of food allergy development and a variety of genes, including those involved with IgE, cytokines, barriers, antiproteases, and pattern recognition. Therefore, offspring can inherit genetic and epigenetic alteration patterns that can shape the food allergy response through the silencing and activation of these genes.

### 2.4. The Allergen March

Finally, the allergen march, the natural progression of allergic disease, has been demonstrated through the 85% probability of development of food allergies or asthma or environmental allergies when having eczema [6,31,32,33]. Hence, it is strongly recommended that when a child has severe eczema, peanuts should be introduced cautiously, following proper diagnostic testing [34]. Additionally, it has been demonstrated that the presence of asthma and allergic rhinitis are associated with a lower likelihood of development of food allergy tolerance from childhood to adulthood [5]. The two major hypotheses for this include the damaged skin barrier allowing for absorption of food allergens, and the shared genetic and environmental risk factors described above.

Overall, this combination of genetic and environmental risk factors promotes the growing rate of food allergy in the United States.

## 3. Etiology

As the prevalence of food allergies has increased over the last few decades, so too has the number of hospitalizations due to food-induced anaphylaxis [6]. Therefore, while awareness has improved, fatality due to severe allergic reactions has not, and it is crucial to identify the underlying immune mechanism to identify treatments and interventions.

**Figure 3:**
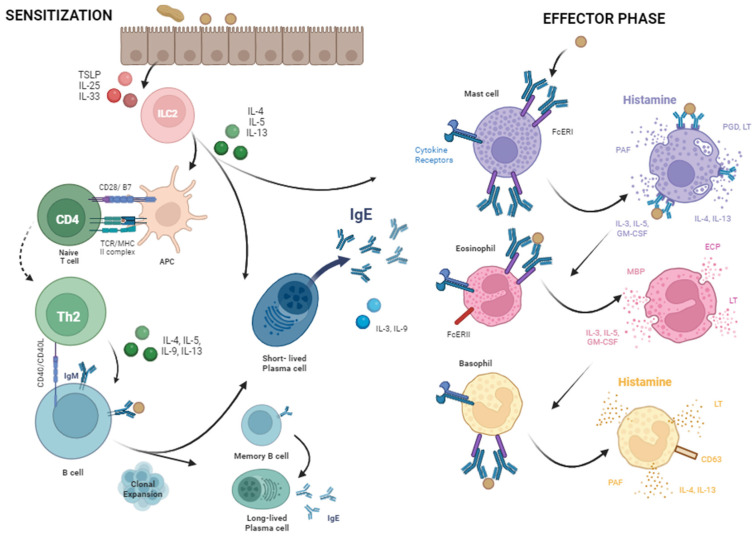
The etiology of food allergies. Food allergy begins with sensitization, whereby the T cells and dendritic cells are primed for an IgE-mediated Th2 response. This Th2 response enables activated B cells to differentiate into IgE-producing plasma cells. Secreted IgE binds its cognate receptor on innate immune cells, including mast cells. These sensitized mast cells can be activated upon re-exposure to the food antigen and release their granule contents. The released molecules, including histamine, are responsible for the range of mild to severe allergic reactions.

A Type I hypersensitivity reaction is an immediate or allergic hypersensitivity to inhaled, injected, or ingested allergens. While the oral mucosa and intestinal tract are constantly in contact with a large variety of food proteins in a daily diet, a relatively small amount are responsible for the allergic reactions observed [7]. Regarding IgE-mediated food allergy, the first exposure of the ingested allergen is typically asymptomatic. Then, with subsequent exposure, the antigen stimulates the immune system and results in an allergic reaction (Figure 3). In non-allergic individuals, the immune system does not perceive these food antigens as harmful, and immunoregulatory Tregs are produced, which act to inhibit an immune response and establish tolerance [7,35]. While food antigens are being sampled, the mucosal immune system instead secretes IL-10 and transforming growth factor beta (TGF-β), through macrophages and dendritic cells, respectively, to promote Treg, IgG4, and IgA production [36,37]. However, allergic individuals produce allergy-specific Th2 cells that can drive an allergic response.

### 3.1. Sensitization

The development of an allergic response involves the allergen first interacting with an epithelial cell, producing alarmins TSLP, IL-33, and IL-25, which drives activation of innate lymphoid cell type 2 (ILC2) and programs dendritic cells to produce Th2-attracting cytokines [38,39,40]. This is achieved by activated ILC2 secreting IL-5, IL-13, and IL-4 [41,42]. When the dendritic cell undergoes maturation and migration to present the antigen to naïve CD4+ T cells in the presence of IL-4, the T cell differentiates into a Th2 CD4+ T cell. A Th2 helper CD4+ T cell expresses CD40 ligand and secretes cytokines IL-3, IL-4, IL-5, IL-9, and IL-13, mediated through transcription factor GATA-3 [9,43].

This cellular Th2 response is what is seen in the context of allergy, through the cytokines secreted by ILC2 and the Th2 CD4+ T cells [44]. Specifically, IL-4 activates B cell proliferation, IL-5 promotes B cell differentiation and recruits eosinophils to the infected tissue and activates them, IL-3 and IL-9 recruit mast cells to the infected tissue, and IL-13 increases the production of epithelial cells in the infected tissue, producing more mucous and increasing their rate of turnover [44]. The Th2 response is stabilized by the IL-4 autocrine and paracrine signaling, as IL-4 also functions in suppressing Treg immunosuppressive functions, and instead reprograms them to also produce more IL-4 [36]. The Th2 cytokine milieu therefore promotes the production of antibodies by the B cell.

When the IgM-expressing B cell first encounters its specific allergen, brought to the lymph node by the dendritic cells, it will be activated under appropriate conditions. Upon uptake and degradation in endocytic vesicles, the peptides are produced and bind to major histocompatibility complex II to be exported and presented to effector CD4+ T cells. As described above, through cytokine secretions and receptor–ligand interactions, the Th2 CD4+ T cell promotes B cells to undergo clonal expansion, resulting in a larger number of B cells that recognize this allergen. Class switching from IgM to IgE is mediated through the high levels of major cytokines such as IL-4 and IL-13 secreted by the Th2 CD4+ T cells and the ILC2 cells, described above [45]. Through interaction of the CD40 ligand with CD40 on the B cell, the B cells differentiate into plasma cells, specifically IgE-secreting plasma cells [45].

Plasma cells are terminally differentiated B cells that are characterized by upregulation of endoplasmic reticulum stress and protein coding genes, which enables a high antibody secretion rate [46]. This triggers the unfolded protein response, maintaining protein homeostasis and quality. Plasma cells are able to also do so by stopping cell cycle progression and preventing DNA replication; therefore, all energy is devoted to antibody production and secretion, regulated by proteins like p18INK4c and epigenetic regulation [46,47]. Differentiation is achieved through the upregulation of plasma cell genes Irf4, Blimp-1, Xbp1, and the downregulation of B cell-specific genes Pax5, Bcl6, and Irf8, to name a few [47]. IRF4 activates Blimp1, which silences B cell lineage transcription factors Pax5 and Bcl6 and activates Xbp1, which coordinates the alterations in cell structure and function, enabling maturation of these antibody-secreting plasma cells [48,49,50,51,52,53].

There are two receptors for IgE, FcERI and FcERII, both recognizing its Fc portion, holding the IgE antibody in a shrimp-like configuration that exposes the antigen-binding site to the environment. This is unique, as other Ig isotypes require antigen encounters first to undergo a confirmational change, exposing the constant region to be recognized by immune cell receptors. With the first exposure, the secreted IgE binds at high affinity, with FcERI receptors present on cells including mast cells, eosinophils, and basophils [45,54].

### 3.2. Effector Phase

An allergic response to the allergen can be generated when re-exposure occurs following sensitization. FcR activation results in Immunoreceptor Tyrosine-based Activation Motifs (ITAMs) signaling following their aggregation. As described above, the first exposure yields a Th2-cytokine milieu high in IL-4, IL-5, and IL-13 secreted by activated ILC2 cells and Th2 CD4+ T cells, which perpetuate the allergic immune response by facilitating the recruitment and activation of these innate immune cells with the IgE receptors. Therefore, the IgE constant region can be bound to its cognate receptors on these cells sensitizing them, as their hypervariable regions are ready to bind and respond to specific antigens upon re-exposure. Sensitization to a specific allergen can also cause cross-reactions to other similar antigens that would constitute re-exposure. This cross-reactivity is why, for example, some individuals allergic to pollen or latex will also react to some fruits and vegetables, which complicates diagnosis and management, as described below [55].

Mast cells are innate immune white blood cells that circulate in the blood and are home to tissues upon maturation, becoming tissue-resident cells [54,56]. As with B cells, mast cells phenotypes are altered by IL-4 and IL-5, largely through receptor expression, proliferation, and activation. Encounter with the specific antigen during re-exposure induces the cross-linking of IgE on the surface of these sensitized cells. This results in mast cell activation and degranulation within minutes, and the release of the pre-made proinflammatory mediators from the cells following fusion of their granules with the cell membrane [54,56].

Mast cell granules contain enzymes, toxic mediators, lipid mediators, and cytokines, which are responsible for the range of mild to severe allergic reaction symptoms [54,56]. Enzymes include tryptases, chymases, matrix metalloproteases, and carboxypeptidases, which help remodel tissues. Toxic mediators include heparin and histamine, which act on and dilate blood vessels. Histamine is the major player in allergy responsible for the increase in vascular permeability and smooth muscle contraction. Lipid mediators in the mast cell granules include leukotrienes and prostaglandins, which take some time to be synthesized following mast cell activation. These lipid mediators also have a vasoactive effect, allowing blood vessel dilation so that cells and molecules can move into the infected tissue site. Additionally, these lipid mediators cause smooth muscle contraction and mucus secretion. Cytokines released include IL-4 and IL-13, which further promote the Th2 response and IgE production. Also, preformed tumor necrosis factor (TNF)-ꭤ is released, which activates the endothelium and allows T cells to extravasate to the infection site and promote inflammation. Cytokines IL-3, IL-5, and granulocyte-macrophage colony-stimulating factor (GM-CSF) mobilize eosinophils from bone marrow. The goal of the mast cell mediators is to expel multicellular parasites through the effect on the gastrointestinal tract and airways [56]. However, in the context of allergy, this inappropriate response causes massive inflammation that can become life-threatening.

While mast cells begin this immune response, eosinophils are the major mediator of the immune response against parasitic infections and the dysregulated immune response seen in chronic allergies [54,56,57]. Eosinophils cooperate with mast cells to produce an allergic response through their toxin and cytokine release upon activation that constitute the late-phase response [54,57]. This is because the chemotactic factors secreted will recruit other inflammatory cells and mediators to the tissue. While FcERI is expressed on eosinophils, its aggregation is not associated with its activation, but other cell surface molecules are, largely cytokine and chemokine receptors, including IL-5, IFN-Y, and IL-16.

Eosinophils release enzymes including eosinophil peroxidase and collagenase, which trigger histamine release from mast cells and tissue remodeling, respectively. Eosinophils also release toxic proteins, including major basic protein, which is toxic to parasites and mammalian cells, and eosinophil cationic protein, which is toxic to parasites. Eosinophils also release cytokines and lipid mediators [54]. Cytokines include IL-3, IL-5, and GM-CSF, which amplifies eosinophil production in the bone marrow, and CXCL-8, which is a chemokine for macrophages. IL-5 can also promote hyper-eosinophilia, which can cause the release of endocardium-susceptible toxic molecules that cause heart damage. Lipid mediators include leukotrienes C4 and D4, which increase vascular permeability, mucus secretion, and smooth muscles contraction. Additionally, platelet activating factor, a chemoattractant for leukocytes, is released. The release of major basic protein and cytokines IL-3, IL-5, and GM-CSF from mast cells and eosinophils also results in basophil activation. Basophils are the third cell type that work in a coordinated fashion with these cells to provide protection from multicellular parasites [54].

Basophils share many characteristics with mast cells; however, they are found in circulation while mast cells are in the tissue. Basophils constitutively express FcERI receptors and contain toxic mediators similar to mast cells and eosinophils [57]. This includes immediate, pre-made inflammatory mediators, such as histamine and the later secretions such as newly synthesized leukotrienes, chemokines, and cytokines. Basophils, eosinophils, and mast cells work together in the initiation and propagation of the immediate and late-phase allergic immune response.

### 3.3. Immunological Memory

With the upgraded immune response that comes with the activation of the adaptive immune system, immunological immunity is generated. Immunologically, this enables a quicker and more robust response before symptoms appear when re-exposed, hence the rationale for vaccinations. Memory Th2 and memory B cells specific to their antigen are generated in both regulated and dysregulated immune responses and are suggested to be critical players in the lifelong persistence of allergy through IgE secretion [58]. Thus, repeated exposure continues to boost the immune response and increases allergen-specific T cells and B cell IgE production.

IgM, IgG, and IgA antibodies are known to develop memory through the establishment of quiescent memory B cells that can be re-activated and long-lived plasma cells that continue to secrete antibodies, mediated by the affinity maturation and class switching discussed above [59]. Memory B cells can respond rapidly and vigorously, constituting the secondary immune response that is stronger and more specific. The memory B cell lifespan has been demonstrated to vary greatly, but this still could play a role in lifelong allergic responses [60]. Furthermore, plasma cells’ lifespans range from days to decades, thought to be largely due to the microenvironment in the bone marrow and other specialized niches supporting their survival. Genes associated with bone marrow homing and antiapoptosis are largely upregulated in these long-lived plasma cells (LLPCs) [46]. These allergen-specific LLPCs are characterized by decreased migration and proliferation halt, deliberately stopped in the G1 phase and preventing DNA replication in the S phase, so that energy is focused on antibody production. LLPCs are also characterized by downregulation of characteristic B cell markers like CD20 with an upregulation in CD138, B cell maturation antigen (BCMA), and Blimp-1, largely regulated by interferon regulatory factor 4 (IRF4) [46,61]. However, this is in stark contrast to plasma cells of the draining lymph nodes, whereby these B cell differentiation and prosurvival genes are not as highly expressed [46]. Therefore, this continuous antibody secretion by LLPCs homing in the bone marrow may also contribute to lifelong allergic response. This phenomenon of immunological memory in the case of IgE-mediated lifelong allergy warrants further investigation.

## 4. Diagnosis

Diagnosis of food allergy combines the history of symptoms, physical exams, and in vitro or in vivo testing.

### 4.1. Symptoms

**Figure 4:**
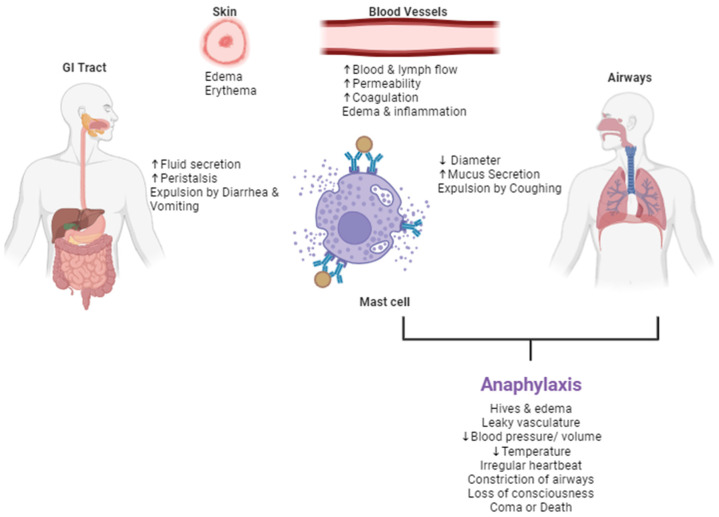
The systemic effects of the IgE-mediated Th2 response to food allergens. The factors secreted through degranulation of the mast cells, eosinophils, and basophils results in systemic effects on the GI tract, skin, blood vessels, and respiratory system. Mild symptoms include mechanisms of expulsion of the allergens from the GI tract and airways. More severe reactions lead to the near-fatal and fatal anaphylaxis, whereby blood pressure and body temperature drops, airways constrict, and this can lead to unconsciousness, coma, or death.

Symptoms of allergic reactions depend on where histamine and other inflammatory mediators are released, how much antigen exposure occurred, and how sensitive the individual is. For example, an individual with a lot of mast cells coated with IgE will be more sensitive to a smaller quantity of food antigen. This allergic reaction is immediate, developing within minutes to two hours after exposure to the food, largely due to the preformed histamine in the immune cell granules. Food allergies can cause mild symptoms such as tingling or itching mouth, hives, swelling, wheezing, vomiting, diarrhea, and abdominal pain. In severe and systemic cases, anaphylaxis can occur because of the allergens entering circulation (Figure 4).

The vasodilation induced by histamine causes swelling, pain, and redness of inflammation. Histamine is also largely responsible for the symptoms of anaphylaxis [30]. With systemic anaphylaxis, the airways become constricted, the throat becomes swollen, the pulse becomes rapid, and shock develops with the blood pressure and temperature drop. This can lead to dizziness and unconsciousness, and, if untreated, can lead to coma or death [62]. Evidence suggests that over 40% of children and 50% of adults with food allergies have experienced this type of severe immune system reaction [63,64].

### 4.2. Diagnostic Testing

Diagnostic testing of allergy has an immunological basis. The three main diagnostic tests for allergic disorders include skin prick tests, blood tests, and oral food challenges. However, the abundance of homologs, for example, in edible foods and aeroallergens, can complicate the results of diagnostic tests and affect food allergy management [55]. These cross-reactive proteins can result in patients’ positive tests to biologically related foods, but not exhibit clinical reactivity [55].

The skin prick test (SPT) assesses mast cell activation through the placement of diluted food extract on the skin. The skin is scratched with a needle and the development of a wheal indicates a possible allergy. These tests, however, generally have good negative predictive value but poor positive predictive value [65].

Blood tests check for levels of total IgE in the blood, normally in the 50–100 μg/L range and specific IgE (sIgE) to an allergen, normally less than 0.1 μg/L. This is assessed through a non-competitive immunometric assay whereby serum is added to an anti-IgE-coated surface that binds the Fc region of IgE and links the antibody to an enzyme or fluorophore. sIgE levels are assessed similarly; however, the specific allergen coats the surface that is bound by the allergen-specific IgE. IgE levels can be monitored annually to see if there are any alterations in specific IgE levels [66]. Peanut (*Arachis hypogaea*) allergen, Ara h 2, is the most important peanut allergen according to the World Health Organization. Ara h 2-sIgE is one example of a blood test serving as an excellent clinical predictor for peanut allergy, with higher specificity compared with whole peanut sIgE measurement and SPTs [67,68]. However, there is a low specificity, as false positives do occur for both whole peanut sIgE and SPT [69].

Oral food challenges (OFCs) occur in a clinical setting, whereby symptoms are intentionally triggered with a small dose of allergen in a well-controlled environment [70]. This includes open challenges whereby the food is administered in a normal serving and the individual is observed for 2 or more hours after. A single-blind oral food challenge involves the test food being hidden in another food that the individual is known to tolerate, controlling for any anxiety reactions or placebo effect. A double-blind placebo controlled food challenge (DBPCFC) removes all bias of expectation from everyone, including the person monitoring the challenge. However, this still carries risk. Additionally, patients may defer an OFC due to confounding medical conditions and medications that may interfere with allergic reactions [70]. New technology using sequential (linear) epitope-specific IgE was able to identify cumulative tolerated doses during DBPCFCs, and, therefore, may be a future surrogate [71]. To further combat the limitations of SPT and sIgE, wherein the extracts are composed of multiple components of the food allergen, with a majority being diagnostically irrelevant, component-resolved diagnostics (CRDs) were developed. CRDs utilize recombinant or purified allergens to recognize the specific molecular epitopes that are targeted by the specific IgE; therefore, they help guide diagnosis and prognosis while eliminating cross-reactivity [72]. However, given its allergen depth, cost, and optimization, OFC remains the gold standard.

The development of cellular testing has demonstrated higher sensitivity and specificity. This is imperative as most mediators like histamine degrade quickly and, therefore, cannot be measured in the clinical setting. The basophil activation test (BAT) measures the expression of activation markers on basophil surface ex vivo through flow cytometry in the presence or absence of food allergens in vitro. These markers include CD63, CD69, and CD203c [38,73]. The BAT has demonstrated more reliability, being more reproducible and requiring few resources and having less risk, over sIgE and SPT [74]. This diagnostic test, however, is not as accessible, as it requires live cells from fresh whole blood and a flow cytometer. But, if the clinical settings permit this, it is a good option in combination with traditional testing as it shows high specificity and positive predictive value [6,75,76]. Mast cell activation tests (MATs) are similar, also appearing to be a promising approach. While performing similarly, MATs are beneficial in that, as opposed to the BAT, they allow the utilization of stored plasma. The other advantage of both the BAT and MATs is the strong correlation with reaction severity that is not seen with sIgE levels and SPT [6]. Further elucidation of the molecular mechanism of food allergy will enable the identification of diagnostic and prognostic biomarkers for severity and treatment of food allergies.

### 4.3. Natural Course

As described above, many of the common food allergies will emerge at infant and toddler ages, with tolerance occurring in early to late childhood. Therefore, it is important to understand this natural course of food allergy for clinicians to guide usage and cessation of elimination diets. The described diagnostic tests can provide insight into the likelihood and timing of resolution of the food allergy and provide important nutritional and quality of life benefits [4,77]. Historically, milk allergy is the first most common, with onset in infancy and high rates of resolution by early childhood. However, heterogeneity has been demonstrated, with rates of tolerance varying by region [4]. Then, with the introduction of solid foods, egg allergy increases in prevalence and becomes the most common in infants, but also tends to resolve in early childhood, with tolerance around 50% in most regions before 5 years old [4]. By preschool age, a similar emergence and resolution trend of wheat allergy is seen [4]. Unfortunately, with peanut, tree nuts, fish, and shellfish allergies, development occurs later and the rate of resolution is much lower, with rates ranging from 4 to 30% by adolescence [4]. Evidence has also begun to suggest shifts to this natural course of food allergies, with some foods that historically resolve early now persisting through adolescence and adulthood [4,5]. This further emphasizes the need for novel therapeutic and preventative options for food allergies.

## 5. Therapeutics and Challenges

Western medicine focuses largely on avoiding triggers, using corticosteroids and other medications, and immunotherapy to combat this allergy pandemic. To aid avoidance, the US Food and Drug Administration (FDA) requires labeling of the nine major foods and food groups (egg, milk, PN, soy, wheat, treen nuts, fish, shellfish, and sesame) [78]. Prompt recognition of allergy symptoms is imperative as complete avoidance is stressful, anxiety-inducing, and can fail. Avoidance is unreliable, with accidental exposures causing 200,000 annual emergency room visits [79,80]. With that, fears of reactions undermine individual and family quality of life, and, therefore, avoidance cannot be the only option [81,82,83,84]. Currently, oral antihistamines, antileukotrienes, steroids, and intramuscular epinephrine are utilized for unspecific symptom management upon exposure [69]. Antihistamines and glucocorticoids do not prevent the progression of an allergic reaction, nor do they treat anaphylaxis as epinephrine does. Thus, Western medicine does not aim to cure or prevent it, just alleviate or improve symptoms. Aside from avoidance and rescue medication, treatment and preventive interventions are limited. Therefore, there is a significant need for more treatments to be developed with a better understanding of the allergic process itself. Progress in uncovering food allergy etiology has aided in the development of therapies to reduce allergic reactions with accidental exposure. Largely, the focus has shifted from broad systemic immunosuppressive drugs to targeted treatment options. New treatments under investigation include antigen-specific immunotherapy, biologicals, and Chinese herbal therapy [69].

### 5.1. Immunotherapy

Food-allergen-specific immunotherapies work through food desensitization. Food allergy oral immunotherapy (OIT) is a specialized treatment whereby the body builds tolerance to the food allergen, decreasing the likelihood of an allergic reaction from accidental ingestion [21]. Mechanistically, OIT induces a shift from Th2 to T regulatory and B regulatory responses that decrease effector cell reactivity and, hence, the allergic response. OIT is given in a clinical setting and begins with a small allergen dose and gradually increases the dose until a target dose is reached over a few months. Palforzia is specifically used to reduce the frequency and severity of peanut allergy symptoms. However, administration is difficult, with the patient having an inability to speak for 2 h. OIT has resulted in a large portion of participants demonstrating symptoms, which are generally mild, but moderate–severe symptoms can occur, and about 10–20% of participants drop out of clinical trials [85]. Hence, substantial therapeutic gaps exist, including reactions, GI inflammation, ineffectiveness for severe cases and for adults, and relapses when off therapy [86,87,88,89]. Additionally, OIT may paradoxically increase IgE levels, with significant immune-reaction risk [90,91,92,93,94].

There are two alternatives to oral immunotherapy: sublingual immunotherapy (SLIT) and epicutaneous immunotherapy (EPIT). SLIT is a non-FDA-approved peanut allergy treatment whereby drops of peanut protein extract are placed under the tongue for 2 min before swallowing. SLIT, however, is less effective than OIT [95]. EPITs work through the delivery of allergens through a patch. The EPIT Viaskin Peanut is also a non-FDA-approved peanut allergy treatment [96]. It is called the “peanut patch”, as it works by providing a tiny dose of peanut allergen to desensitize the patient to peanut allergens. EPIT treatment results in increased IgG4 levels and IgG4/IgE ratios and reduced Th2 cytokines and basophil activation [97]. The downside is that it appears that a longer treatment duration is required to demonstrate the same outcomes of OIT [95]. While OIT has better efficacy, it appears that some patients may develop eosinophilic esophagitis [38]. Unfortunately, over half of successfully desensitized patients lose tolerance after a short period of avoidance [6,86,98,99,100]. In these patients, SPT wheal diameter size returned to baseline levels and had much higher peanut sIgE at baseline and end-of-study when compared to the patients that had sustained unresponsiveness [99]. Similar results were seen in another study, where authors concluded that lower IgE to Ara h 1–3, peanut sIgE, or peanut IgE/IgG4 ratio, or a lower basophil activation test response to peanut at baseline are associated with improved success rates [101]. Another study also saw that when comparing patients that had sustained responsiveness to those that did not, absolute counts and migratory activity of antigen-induced Treg were significantly increased, characterized by high Foxp3 expression [102]. In all, current immunotherapies are largely studied in peanut and milk. Additionally, when comparing OIT to SLIT to EPIT, the efficacy, safety, adverse effects, adherence, and feasibility results in no one option being better than the other two, nor do any present as a permanent cure [103].

In an effort to combat the high risk of anaphylaxis with crude extract introduction as a therapeutic, the development of a novel Ara h 2- Fcϒ fusion protein was developed. It successfully blocked the release of histamine from basophils of patients with allergies upon the introduction of whole peanut extract [104]. Additionally, in humanized transgenic mice, it also inhibited acute anaphylactic reactivity and inflammation induced by crude peanut extract [104]. With the same objective in mind, a vaccine containing modified peanut proteins Ara h 1, 2, and 3 at IgE-binding sites that were encapsulated in heat/phenol killed *E. coli* was investigated [105]. Unfortunately, frequent adverse, including severe, reactions occurred in humans [105]. Similarly, modified (m) Ara h 1–3 was also introduced to heat-killed Listeria monocytogenes (HKLM) as an adjuvant [106]. Mice that were given this treatment demonstrated reduced alterations in core body temperature, bronchial constriction, histamine levels, peanut sIgE, IL-5, and IL-13 levels, with increased IFNg levels [106]. Therefore, this model may be a potential approach for treating peanut allergies. This group also developed another peanut immunotherapy using CpG-coated poly(lactic-co-glycolic acid) nanoparticles containing peanut extract (CpG/PN-NPs) [107]. The treatment itself did not cause anaphylaxis, but protected the mice from anaphylaxis, as evidenced by decreased alterations in body temperature, symptom scores, plasma histamine levels, IgE/IgG1 levels, and an increase in IgG2A levels [107]. The splenoyctes collected were tested and also showed decreased Th2 cytokines, including IL-4, IL-5, and IL-13, and increased IFN-Y levels [107]. Therefore, this mechanism of immunotherapy may be a promising therapeutic strategy.

In a similar mechanistic realm, modifying the food’s allergenicity through introducing food cooked in alternative ways may decrease the risk of the allergic reaction, as opposed to complete avoidance. As described above, in China, peanuts are boiled, while in the United States, peanuts are roasted. However, in both countries, peanut consumption is similar and, therefore, it is thought that boiling may reduce the amount of allergen exposure. One study was able to demonstrate that the boiled peanuts showed a lower sensitization than roasted and raw peanuts, as evidenced by mouse ethology, serology, and pathology, following exposure [108]. For example, physical discomfort was apparent in mice eating raw and roasted peanuts, with diarrhea and weight loss, which was made further evident by the inflammation in the jejunum [108]. Th2-type serological indexes were reduced in boiled peanuts including reduced degranulated mast cells, IgE levels, and IL-4 with increased IFN-Y [108]. They also demonstrated an increased resistance of roasted peanut protein to digestion in simulated gastric fluid and altered structures of the Ara h 2 peanut allergens [108]. In an open-label, Phase 2, single-arm clinical trial, patients underwent OIT involving sequential up-dosing with boiled peanut, and desensitization was successful in 80% of participants [109].

In a similar manner, baked milk products are generally tolerated in milk-allergic children. When comparing milk-allergic children that tolerated baked milk to dose escalation of less heat-denatured forms of milk, immunologic changes, including reductions in IgE and increases in IgG4, were observed [110]. Baked eggs are also increasingly being used for egg allergy, rather than avoidance [111]. Reducing the allergenicity of peanuts and other foods has high potential; however, these methods need to be optimized for full effectiveness and subsequently sustained unresponsiveness [112].

In all, immunotherapy lacks standardized protocols, with various administration routes, doses, timing, etc. Additionally, it runs the risk of adverse reactions, with concerns for both safety and long-term consequences. The induction of long-term tolerance to food allergens has yet to be developed. Furthermore, there is an immunological memory aspect of an allergic response whereby the long-lived plasma cells and memory B cells are generated, which remain unaffected by avoidance and immunotherapies. Therefore, novel strategies to target these persistent cells are critical for curative immunotherapy. Recent attempts to combat this issue are allergen-derived peptide immunotherapy. PVX108 consists of seven peptides of immunodominant T cell epitopes of major peanut allergens Ara h 1 and Ara h 2. This has yielded safe and tolerable responses in Phase 1 clinical trials [113]. Further work may provide evidence that peptides can be used to treat food allergies without adverse effects or eventual loss of tolerance. Clinical trials for the described therapeutics for both individual food and multi-food allergies have been completed, and others are continuing to recruit (Table 1).

### 5.2. Biologics

**Figure 5:**
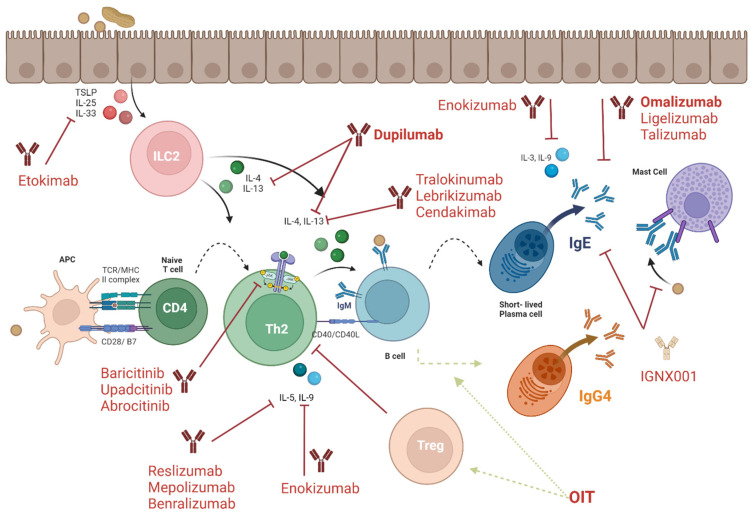
Current therapeutic targets and mechanisms. Largely through the inhibition of alarmins and inflammatory cytokines binding to their receptors on immune cells, including IL-4, IL-13, IL-5, and IL-9, and blocking IgE, biologics are serving as effective therapeutics in the event of an accidental exposure in preventing severe immune responses.

Biologics have demonstrated their promise as a food allergy treatment given the targeting of the cells and pathways of the immune system that result in allergic reactions, not the allergen itself. These drugs are often antibody proteins that bind molecules of these pathways to prevent their activation (Figure 5). Most allergy biologics were approved for other atopic diseases and then investigated for food allergy [114].

#### 5.2.1. Anti-IgE Therapeutics

Anti-IgE therapies are biologics that target and block IgE. Monoclonal antibodies that block IgE function either reduce their free levels or bind to the FcERI and FcERII receptors, preventing an allergic response and stopping inflammation by preventing histamine release. Anti-IgE therapies also function to decrease FcERI expression. There is an older generation IgE monoclonal antibody targeting IgE, talizumab, which demonstrated efficacy in peanut allergy patients; however, there has been a shift to newer-generation biologics, including omalizumab and ligelizumab. Omalizumab (Xolair) was FDA-approved as a food allergy therapy for extra protection for accidental exposure [115,116]. Omalizumab is approved for adults and children 1 year old and older with food allergies. The subcutaneous injection is given every 2–4 weeks and can be performed in a clinical setting or at home. Evidence also suggests that it can be used prior to administering OIT and subsequently improves the safety of OIT and time to reach the OIT maintenance dose [117,118]. Omalizumab efficacy was also seen in a multi-food allergen OIT treatment regimen and in patients with additional allergic diseases, including severe asthma and food allergy [119]. As with OIT alone, sustained unresponsiveness does occur [114,120]. This is likely because omalizumab blocks IgE without targeting the production of IgE [78]. Ligelizumab also targets IgE with a higher affinity for free IgE than omalizumab; however, it did not demonstrate superiority over omalizumab in clinical trials [121].

Other methods for blocking IgE binding to FcERI include designed ankyrin repeat proteins (DARPins) and the coaggregation of fusion proteins to block their interaction [122]. DARPins are proteins with motifs for protein–protein interactions. DARPins have demonstrated a high potential for the inhibition of IgE-mediated allergic reactions through FcERI binding. Current work has not progressed beyond in vivo study whereby safety and efficacy in humans is a high concern. For example, these proteins have a high potential for immunoreactivity. Additionally, blocking all FcERI receptors may have negative, broad, downstream immune consequences. Meanwhile, the coaggregation of fusion proteins takes advantage of the inhibitory signaling of IgG Fc receptor FcYRIIb. As described above, FcERI activation uses ITAM but FcYRIIb utilizes the inhibitory motif (ITIM) [123].

Coaggregation, or fusion, of the FcE and FcY has demonstrated prevention of allergy-mediated anaphylaxis through these bispecific antibodies [124]. Hindrance in the preclinical stage may be due to their limitations, including their short half-life in vivo and their immunogenicity, making it challenging for chronic administration [125,126]. Allergen-specific IgG antibodies have also demonstrated the ability to crosslink the two Fc receptors in the presence of sIgE and the allergen, or competitively inhibit binding of the allergen to its sIgE altogether [127]. Recently, IgGenix announced IGNX001, an IgG4 mAb-based therapeutic for peanut allergy. This candidate competitively inhibited allergic responses by binding to peanut allergens and prevented anaphylaxis in an in vivo mouse model [128]. Taken together, these approaches have the same goal of inhibiting the ability of inflammatory mediators released induce by FcERI and the anaphylaxis mediated by IgE, with some promising success.

#### 5.2.2. Cytokine- and Receptor-Targeted Therapeutics

Beyond blocking IgE from binding its receptors on immune cells, biologics have begun to target proinflammatory cytokine receptors and cytokines that are secreted and mediate the allergic immune response including IL-4, IL-13, and IL-5. Anti-IL-4Rꭤ human IgG4 monoclonal antibody Dupilumab (Dupixent) has emerged as a successful therapeutic strategy, as IL-4R is an attractive target for intervening in severe and chronic allergic responses [122,129]. IL-4 and IL-13 secretion and subsequent binding to their receptor, as described above, plays a major role in the development of the Th2 immune response and driving the pathogenesis of allergy. There are two types of IL-4 receptors (IL-4R), both composed of two subunits, but that share the IL-4Rꭤ. The Type I IL-4R is composed of IL-4R heterodimers, ꭤ and ϒc, that bind to IL-4 [129]. The Type II IL-4R is composed of IL-4Rꭤ and IL-13Rꭤ1 that binds to both IL-4 and IL-13. Binding of these Th2-inducing cytokines triggers a signal transduction cascade, resulting in phosphorylation events of JAK kinases and downstream pathways, including STAT6, PI3k/Akt, and MAPK, and subsequent GATA-3 transcription factor binding and further promoting of IL-4 production [129]. Dupilumab targets the IL-4R complexes by inhibiting the dimerization of the IL-4Rꭤ chain to ϒc or IL-13Rꭤ1 and/or inhibiting IL-4 binding to its receptor. As described above, IL-4 can affect multiple cell types in the allergic response, including ILC2, DC, T, and B cells. Therefore, Dupilumab can prevent B cell activation and IgE secretion and downstream propagation of the allergic response [123,130]. Dupilumab was the first FDA-approved biologic for eosinophilic esophagitis, and in combination with omalizumab, it has been investigated for blocking both IgE and IL-4/IL-13 and is also being investigated as both monotherapy and as an adjunct with OIT for food allergy [130,131,132].

The repurpose of antibodies that improve severe atopic dermatitis symptoms in patients with and without food allergies may warrant investigation for food allergy management due to the similarities in pathogenesis, including the IL-13 antagonists tralokinumab and lebrikizumab [133]. Likewise, the repurpose of antibodies designed for the treatment of eosinophilic esophagitis may also yield positive results in the context of food allergy reactions. Cendakimab is a monoclonal antibody that specifically targets IL-13R. This mAb has been investigated in eosinophilic esophagitis whereby gene expression patterns during treatment correlated with clinical improvements [134]. The anti-IL-5 antibodies, mepolizumab and reslizumab, bind to IL-5 and are also able to mitigate eosinophilic esophagitis through the prevention of IL-5 signaling, reducing the production and survival of eosinophils [135]. Similarly, eosinophils are depleted through IL-5 receptor (IL-5R) targeted mAb benralizumab. In all, the blocking of these major cytokines and binding to their receptors could yield a promising therapeutic strategy for food allergy, given similarities to pathogenesis processes.

JAK inhibitors (jakinibs), baricitinib, upadacitinib, and abrocitinib are oral, FDA-approved drugs for atopic dermatitis and, hence, were also attractive treatment options for repurposing in food allergy. Mechanistically, this decreased allergic response is effective through its widespread effect on downstream signaling, largely decreasing IL-13 release and mast cell activation. Abrocitinib has demonstrated decreased basophil and T cell activation in peanut allergy cases [136]. In all, JAK inhibitors have begun to be investigated as attractive options for monotherapy or in combination with OIT [122]. However, there is minimal information on their use in food allergies, urging more investigation.

As described above, IL-33, IL-25, and TSLP are alarmins that signal a danger response and are largely responsible for the initiation of the IgE-mediated allergic immune response [137]. Therefore, targeting these nuclear cytokines may prevent the downstream promotion of a Th2 response and, hence, inhibit the pathogenesis of allergy. The anti-IL-33 biologic, etokimab, is being studied for peanut allergy therapy, with hopes of tolerizing patients in the event of accidental exposure, through blocking this cytokine from promoting the Th2-response. Early studies demonstrated that etokimab was safe and well tolerated [138]. However, a study in mice demonstrated that the inhibition of all three alarmins was necessary to suppress food allergy [139]. For now, safety and efficacy still need to be determined, as there is the potential for adverse effects.

Many of these therapies, whether as monotherapy or in combination with each other or OIT, still need safety and efficacy studies, while also extrapolating who to treat, dosing, and timing in the context of food allergy [122]. While promising, biologicals entail high cost, injection site pain, acute reactions including anaphylaxis, and indefinite treatment [140]. And, while directly addressing the underlying mechanisms through cellular and molecular interactions, the downstream side effects, including susceptibility to some diseases, for example, warrants investigation. Clinical trials for the described therapeutics for both individual food and multi-food allergies have been completed and others are continuing to recruit (Table 1).

### 5.3. Probiotics

As described above, dysbiosis of the gut normal flora has demonstrated roles in allergy development and, hence, the hypothesis that probiotics may be a viable therapeutic option for tolerance induction [141]. This unbalancing of the gut normal flora is due to a plethora of factors including diet, medication, and the immune system. It is apparent that the gut microflora can play a role in barrier protection but also immunomodulation. These commensal bacteria largely function in immune tolerance through interaction with the gut mucosal immune system and promoting or suppressing the Th1 and Treg cells or Th2 cells and IgE production [142].

The normal gut flora is typically characterized by vast diversity and dominated by *Proteobacteria*, *Firmicutes*, *Bacteroidetes*, and *Actinobacteria*. Thus, changes in gastrointestinal microbial composition and patterns have been measured in stool and characterized by specific food allergen sensitizations [143,144,145]. In mice, an allergy-associated microbiota can drive an allergic phenotype in germ-free mice when transplanted from mice or humans [146,147]. Therefore, targeting the gut microbiome through its reconstitution with a fecal microbiota transplant and the introduction of normal, healthy, commensal bacteria or probiotic bacteria in combination with OIT are being investigated [143,148]. In fact, the combination demonstrated long-lasting benefit and persistent suppression of the allergic response in peanut allergies [149]. Fecal microbiota transplants in mice have largely yielded protective outcomes to food allergies [150]. Current work is underway to evaluate the safety and efficacy of fecal microbiota transplantation on peanut allergy in patients (NCT02960074), but more work still needs to be carried out. In a similar mechanistic hypothesis, maternal vaginal microbiota introduction in neonates is being investigated (NCT03567707, NCT03928431) (Table 1).

**Figure 6:**
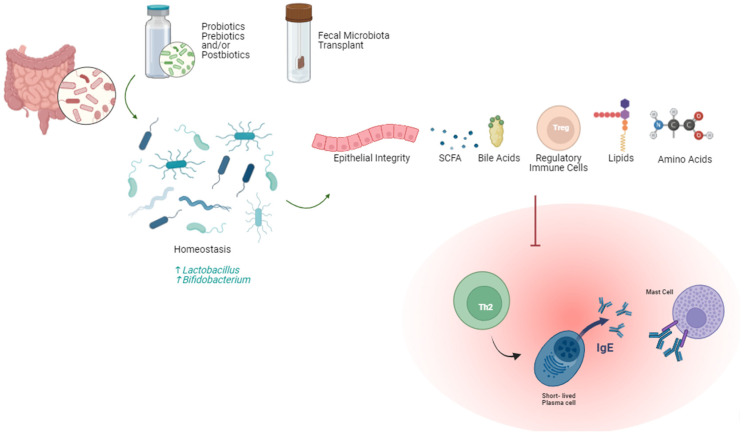
Current probiotics mechanisms to reduce symptoms of food allergies.

Probiotics are beneficial microorganisms that can colonize the gut and improve homeostasis and, hence, promote the beneficial effects of the normal flora. Through changing the normal flora structure upon colonization, probiotics can both antagonize harmful bacteria and affect the metabolism of the beneficial normal flora bacteria (Figure 6). *Clostridia* and *Bifidobacterium* gut microorganisms can attenuate allergic responses by promoting Breg or Treg cells that inhibit Th2 CD4+ T cells, or by inhibiting the allergen absorption [38,151,152,153,154]. Additionally, *Lactobacillus* species has demonstrated usefulness as a supplementation for OIT, sustaining desensitization [155,156,157,158]. Further, gut flora dominance of *Firmicutes*, *Ruminococcaceae*, and *Lachnospiracaea* is evident in infants with allergies [159]. This highlights the overall reduction in microorganism diversity seen in children with various food allergies. Through metabolism modulation by prebiotics, it is possible to affect the production of bile acids, short-chain fatty acids, lipids, and amino acids [160]. Largely, downstream effects include modulation of the Th1/Th2 balance and mitigation of the immune-cell-mediated inflammation that would otherwise occur. This also results in improved epithelial barrier function, which can prevent an inflammatory response.

There are some limitations of probiotic use, including the potential adverse effects through interference with the normal gut flora, including overstimulation of the immune system and microbial resistance. This risk is significantly higher in immunocompromised individuals, whereby the introduction of foreign bacteria may result in adverse effects, including sepsis [151]. Additionally, some probiotics are unstable at room temperature, yielding a short shelf life and short life of bioreactivity [151]. To combat this, investigations of pre- and postbiotics have also been investigated [161]. Postbiotics are microbial or component inhibitors with immunomodulatory effects, largely anti-inflammatory, antitumor, and antioxidant, through mediation of cytokines, promoting gut homeostasis and reducing inflammation. While demonstrating a minimization of risk compared to ingesting live bacteria, postbiotics have not demonstrated the same success as live bacteria in probiotics but may be considered for future investigation as an adjuvant therapy [162]. Current work is also investigating prebiotics in conjunction with immunotherapy, including low amylase maize starch (LAMS), a prebiotic dietary fiber, and oligofructose [163]. Thus, an increase in microflora diversity through the administration of cocktails of these protective bacteria or prebiotic or postbiotic supplementation urges further investigation in clinical trials.

### 5.4. Natural Products

The recurring theme of the above therapeutic approaches is the urgent need for continued clinical investigation to identify therapies that are lifesaving and lasting. Naturally occurring compounds have demonstrated immunomodulatory properties and, hence, have the potential to be utilized as therapeutic options in diseases like cancer, diabetes, and asthma. These compounds have been reported to modulate allergic responses, serving as both preventative and treatment options [164].

Traditional Chinese medicine (TCM) is an attractive therapeutic strategy, as, generally, they are effective, low cost, and produce fewer side effects. Food allergy herbal formula-1 (FAHF-1) and FAHF-2 have been extensively investigated for their role as a therapeutic, demonstrating suppression of clinical symptoms through modulation of the above described pathogenesis of food allergy. The 9-herb botanical compound, FAHF-2, successfully inhibited IgE production and had long-lasing beneficial effects on T and B cells in vivo. Specifically, mouse models utilizing FAHF-2 prevented histamine release and anaphylaxis, with decreased peanut-specific IgE, Th2 cytokines expression including IL-4, IL-5, and IL-13, and mast cell degranulation, and coincided with the increase in peanut-specific allergy-protective IgG2a and cytokine expression including IFNY and IL-10 [165,166,167,168]. The switch from an allergic to non-allergic phenotype was confirmed in in vitro human cell lines, whereby IL-5 was reduced and IL-10 and Tregs were induced in allergen-stimulated PBMC cultures [169]. Phase 1 clinical trials demonstrated that FAHF-2 doses were tolerable and exhibited long-term safety [170]. The Phase 2 clinical trials demonstrated that ex vivo stimulation decreased basophil activation markers [171].

Originally, the daily dose was high, but an ethyl-acetate and butanol purified FAHF-2 (EBF2) was developed [172]. This enhanced formula showed similar results, with anaphylaxis protection and reduction in IgE, but also demonstrated gut microbiome modulations in mouse models [172]. Mechanistically, EBF2 demonstrates a non-toxic dose-dependent reduction in IgE production and murine macrophage TNF-ꭤ production [173]. An EBF2 triple therapy Phase 2 clinical trial protocol involving the randomization of EBF2 and placebo in conjunction with 4 months of omalizumab and the introduction of multi-OIT to three food allergens demonstrated that outcomes were not statistically different between the two treatments [174].

**Figure 7:**
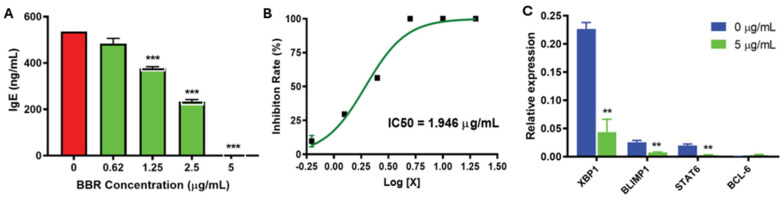
The use of natural products for food allergy therapeutics validated in vitro, in vivo and ex vivo. Adapted with permission from ref. [172]. BBR dose-dependently inhibits the IgE expression by U266 IgE-expressing myeloma cells (**A**) at an IC_50_ dose of 1.946 μg/mL (**B**). This coincided with a reduction in genes that are associated with plasma cell activation (**C**). BBR = berberine. ** *p* < 0.01; *** *p* < 0.001 vs. untreated. N = 3 independent culture.

168Identification of the active compounds in the TCM responsible for the prevention of food allergy responses revealed a major ingredient of FAHF-2: berberine. In mouse models, berberine induced sustained IgE reduction and long-term tolerance to peanuts in mice with peanut allergy. Mice administered an oral berberine-containing natural medicine alluded to its efficacy in food allergy treatment with benefits associated with distinct microbiota. Berberine was also able to stop the production of IgE in food allergy patients’ PBMCs and, in a non-toxic, dose-dependent manner, reduce mast cell degranulation [175]. IgE production by B cells decreased (Figure 7A,B). This is associated with regulators of IgE isotype switching and FcER1 early signaling. Further, mechanistically, berberine works to suppress transcription factors involved in the maintenance of long-lived plasma cells, Xbp1 and STAT6 (Figure 7C) [172]. In all, this suggests berberine’s value for food allergies and other mast cell disorders.

There are other TCM products that have been investigated in less detail. This includes resveratrol, turmeric, Red Ginseng extract, and polyphenol-enriched apple extract, which have also demonstrated food allergy attenuation through balancing of the Th1 and Th2 phenotypes in vivo [176,177,178,179].

Novel botanical compounds have gone through multiple rounds of development from initial formulation to purification, and further identification of the specific ingredients that are the active compounds that target this IgE-mediated food allergy response. These formulations and compounds as monotherapy or in conjunction with current therapeutics are promising, as the anti-inflammatory mechanisms can be specific and broad, targeting the production of many cytokines at once. Additionally, their observed and studied safety profile promotes early use, even in infants, with monitoring every 6 months to one year. Further research is crucial for the utilization of these potentially life-altering and lifesaving compounds for patients with severe food allergies.

Clinical trials for the described therapeutics for both individual food and multi-food allergies have been completed, and others are continuing to recruit (Table 1). This includes a combination of immunotherapy, biologics, natural products, and/or probiotics. Preclinical data have demonstrated great promise for natural products and probiotics in the prevention and treatment of food allergies, so hopefully the clinical data mirror this, enabling these novel therapeutics to narrow treatment gaps in food allergy.

## 6. Conclusions

A plethora of research has been carried out into the elucidation of the etiology of IgE-mediated allergy, whereby the immune response that would be required in the context of parasites and environmental toxins is misemployed against harmless allergens. This is possibly a result of the uneducated/dysregulated immune system brought about by Western culture, through diet, hygiene, and epigenetics that disrupt both the normal microbiota and immune response. In individuals without allergies, the introduction of an allergen to the body would result in regulatory signaling, whereby the body would not mount an inflammatory immune response. However, allergic individuals, when exposed to an antigen, prime the immune system for a robust Th2 CD4+ T cell response that sensitizes the body for massive inflammation upon repeated exposures, resulting in mild to severe allergic reactions. These reactions can be life-threatening, hence the urgent need to uncover the mechanism of food allergy to develop targets that will alleviate its progression. Current therapeutic strategies largely constitute avoidance and rescue upon accidental exposure, which can diminish quality of life. Therefore, prophylactic measures, including immunotherapies, and therapeutic strategies, including biologics that target specific molecules and pathways that promote this response, have been investigated. Currently, there are drawbacks to these measures, including the potential adverse effects and the re-sensitization that comes when stopping the therapy. Homeostasis of the normal flora to instead promote the regulatory response and restore tissue integrity through pro-, pre-, and postbiotics, and fecal microbiota transplant have been explored. This, however, comes with high risk as well. Therefore, the more effective, affordable, and safe TCM compounds have become an attractive avenue for study. These natural compounds have demonstrated promising results in both efficacy and improved quality of life for people with food allergies.

## Figures and Tables

**Table 1:** Current clinical trials for therapeutics of food allergy, by type.

Number	Treatment	Condition	Status
**Biologics**			
**NCT02643862**	Omalizumab	Multi-food allergy	Completed
**NCT01157117**	Omalizumab + OIT	Cow milk allergy	Completed
**NCT02920021**	Etokamib	Peanut allergy	Completed
**NCT03881696** **NCT01510626**	Omalizumab + OIT	Multi-food allergy	Active, not recruiting
**NCT03682770**	Dupilumab + OIT	Peanut allergy	Completed
**NCT04148352**	Dupilumab + OIT	Cow milk allergy	Recruiting
**NCT00932282**	Omalizumab + OIT	Peanut allergy	Completed
**NCT04943744**	Omalizumab + Dupilumab + OIT	Multi-food allergy	Enrolling by invitation
**Immunotherapy**			
**NCT00932828** **NCT04222491** **NCT00597675** **NCT01814241** **NCT03907397** **NCT01867671** **NCT00815035** **NCT01324401** **NCT02203799** **NCT01750879** **NCT03736447** **NCT01891136** **NCT03251508** **NCT04603300** **NCT02635776** **NCT01274429** **NCT01074840** **NCT02993107** **NCT01987817** **NCT03292484** **NCT03352726** **NCT02198664** **NCT03126227** **NCT03337542**	OIT	Peanut allergy	Completed
**NCT05621317**	OIT	Peanut allergy	Recruiting
**NCT04090203**	OIT	Peanut allergy	Active, not recruiting
**NCT00461097** **NCT04056299** **NCT01846208**	OIT	Egg allergy	Completed
**NCT00429429** **NCT00597727** **NCT01373242** **NCT00580606** **NCT02304991** **NCT02991885**	SIT	Peanut allergy	Completed
**NCT05440642**	SIT	Peanut allergy	Recruiting
**NCT02350660**	OIT	Peanut and mammalian meat allergy	Completed
**NCT04856865** **NCT01490177**	OIT	Multi-food allergy	Completed
**NCT03504774**	OIT	Shrimp and cashew allergy	Terminated
**NCT03181009**	Multi-OIT	Multi-food allergy	Completed
**NCT01904604** **NCT01170286** **NCT01675882** **NCT02636699** **NCT01955109**	EIT	Peanut allergy	Completed
**NCT05741476**	EIT	Peanut allergy	Recruiting
**NCT05424731**	EIT	Peanut allergy	Available
**NCT03211247**	EIT	Peanut allergy	Active, not recruiting
**NCT03859700**	EIT	Peanut allergy	Recruiting by invitation
**NCT02223182** **NCT02579876**	EIT	Milk allergy	Completed
**NCT00732654**	OIT + SIT	Milk allergy	Completed
**NCT01162473** **NCT00465569** **NCT03462030**	OIT	Milk allergy	Completed
**NCT01084174**	OIT + SIT	Peanut allergy	Completed
**NCT01980992**	OIT	Wheat allergy	Completed
**NCT0546753**	OIT	Tree nut allergy	Completed
**Natural Products**			
**NCT00602160**	FAHF-2TM	Multi-food allergy	Unknown
**NCT02879006**	EB-FAHF-2, Multi-OIT and Omalizumab	Multi-food allergy	Unknown
**Probiotics**			
**NCT05138757**	Prebiotic + OIT	Peanut allergy	Recruiting
**NCT03936998**	Postbioitc + Antibiotic + OIT	Peanut allergy	Recruiting
**NCT00664768**	Prebiotic + Probiotic	Milk allergy	Completed
**NCT02960074**	Fecal microbiota transplant	Peanut allergy	Completed

Accessed September 2024.
